# The value of urea, creatinine, prolactin, and beta sub-unit of human chorionic gonadotropin of vaginal fluid in the diagnosis of premature preterm rupture of membranes in pregnancy

**DOI:** 10.4274/tjod.48902

**Published:** 2016-06-15

**Authors:** Marzieh Ghasemi, Reyhaneh Jaami, Ashraf Alleyassin, Alireza Ansarimoghaddam

**Affiliations:** 1 Zahedan University of Medical Sciences, Reproductive Health Research Center, Zahedan, Iran; 2 Zahedan University of Medical Sciences, Ali-Ibn Abitaleb Hospital, Clinic of Obstetrics and Gynecology, Zahedan, Iran; 3 Tehran University of Medical Sciences, Shariati Hospital, Clinic of Obstetrics and Gynecology, Tehran, Iran; 4 Zahedan University of Medical Sciences, Department of Epidemiology and Health, Zahedan, Iran

**Keywords:** Premature preterm rupture of membranes, urea, creatinine, Prolactin, β-hCG, vaginal fluid

## Abstract

**Objective:**

To evaluate the effectiveness of urea, creatinine, prolactin, and the beta sub-unit of human chorionic gonadotropin (β-hCG) of vaginal fluid in the diagnosis premature preterm rupture of membranes (PROM).

**Materials and Methods:**

In this observational study, 160 pregnant women with gestational age of 28 to 40 weeks were divided into two equal groups: investigation (documented PROM) and control (intact membrane) groups. Five cubic centimeters of normal saline was poured into the vagina of all participants and the liquid was extracted after a few minutes using a syringe. The liquid was sent to a laboratory for examination. Data were analyzed using a t-test.

**Results:**

The volume of urea, creatinine, prolactin, and β-hCG was significantly different in the two groups (p<0.001). Based on receiver operating characteristic curve and cut-off point, sensitivity, specificity, positive and negative predictive values of β-hCG for detecting PROM were 87.5%, 86%, 86.4%, and 87.3%, respectively. Also, the same factors for urea in detecting PROM were 79.7%, 82.5%, 81.8%, and 80.4%, respectively. Creatinine had 74.6% sensitivity, 85% specificity, and 83% and 77.2% positive and negative predictive values for detecting PROM. Finally, prolactin had 87.5% sensitivity, 90% specificity, and 90% positive and 88% negative predictive values for detecting PROM.

**Conclusion:**

Prolactin and β-hCG have more diagnostic value than urea and creatinine in detecting PROM, and can be used in suspected cases.

## INTRODUCTION

Premature rupture of membranes (PROM) refers to fetal membranes’ rupture before the onset of labor. If it occurs before 37 weeks of pregnancy, it is called premature preterm rupture of membrane (PPROM)^(1)^. In term or preterm pregnancies, a long duration between PROM and delivery can result in maternal and neonatal morbidity. This includes intrauterine infections (chorioamnionitis), neonatal and fetal sepsis, fetal prematurity, placental abruption, umbilical cord prolapse, cesarean delivery, and an increased risk of maternal and neonatal mortality^(1,2,3,4,5,6)^.

Any patient with a history of vaginal leakage during pregnancy or a decreased level of amniotic fluid in ultrasound should be evaluated carefully because of the adverse effects on pregnancy outcomes. Early and accurate diagnosis allows clinicians to design some interventions for optimizing maternal and neonatal outcomes and decrease serious complications^(7)^.

Detecting PROM is sometimes easy in a speculum examination with the observation of amniotic fluid accumulation in the vagina or liquid outflow from cervix^(8)^. However, when the membrane rupture is small or it is impossible to clearly see amniotic fluid leakage, PROM cannot be detected easily, which might lead to failure in diagnosis and non-performance of necessary interventions^(9,10)^. There are a few methods for PPROM diagnosis. Fern and nitrazine are two traditional, commonly used tests. Although they are easy and rapid tests, both have high false positive and negative results, for example through blood, semen or cervical mucus contamination or technical errors, which means they are not completely reliabe^(8,9,10,11,12,13)^.

Ultrasound examination with amniotic fluid determination is not a good test because it cannot differentiate PROM from other causes of oligohydramnios^(7)^. Although the amnio-dye or tampon test is a standard test for accurate diagnosis, it involves amniocentesis and instillation of dye; therefore, it is an aggressive test and has a risk of placental abruption, miscarriage, bleeding, infection, and iatrogenic uterine perforation^(7)^. The Amnisure ROM test is another new test that is easy, fast, and minimally invasive, with high sensitivity and specificity. This test identifies trace amounts of placental alpha-microglobulin-1 (PAMG-1), which is abundant in amniotic fluid^(14,15)^. However, Amnisure it is not available in many centers and it is expensive.

For this reason, a non-invasive, simple, and inexpensive method of detecting PPROM is required. Several markers have been studied such as alpha-fetoprotein, fetal fibronectin, creatinine, insulin growth factor binding protein 1, urea, prolactin, and β-hCG^(5,7,13,14,15,16,17,18)^.

β-hCG is a glycoprotein that is secreted in the placenta from syncytiotrophoblasts. Prolactin is a single-chain polypeptide that is secreted during pregnancy from the mother’s and fetus’s pituitary and decidua. Urea and creatinine are both excreted through glomerular filtration. These markers are also available in amniotic fluid and have been examined for finding PPROM in some studies^(5,16,17,18)^. The present study evaluated the value of urea, creatinine, prolactin, and β-hCG of vaginal fluid in the diagnosis of PPROM in pregnancy.

## MATERIALS AND METHODS

Between April 2013 and August 2014, 160 pregnant women with gestational age of 28 to 40 weeks were enrolled in the study. All women presented to our center in Zahedan, Sistan and Baluchestan province, Iran. The aim of the study was explained for all participants before their participation and informed consent was received. The study was Approved by the Ethics Committee of Zahedan University of Medical Sciences. All patients were divided into two groups. The PROM group comprised women with ruptured membranes and the control group included women who had just presented to our center for periodic examinations. The mean ages of the investigation and control groups were 25.0±6.5 years and 25.8±5.5 years, respectively (p=0.386).

Gestational age was determined based on the last menstruation period and ultrasound of the first trimester of pregnancy. Membrane rupture was verified in a sterile speculum examination and observation of fluid leakage in the cervix or accumulation of fluid in the posterior fornix of the vagina, or by both nitrazine and Fern tests. Patients with fetal malformations, fetal growth restriction, fetal distress, placenta previa, vaginal bleeding, vaginal infection, maternal disease, hypertension, preeclampsia and other pregnancy complications were excluded.

Five cubic centimeters of normal saline sterile solution was poured by a syringe in all participants posterior vagina fornix. After a few minutes the fluid was aspirated by the same syringe and was sent to a laboratory for examination. The liquid was centrifuged for 10 minutes. The Alcyon automatic biochemical kit was used to measure urea and creatinine (Pars co., Iran) and DiaPlus enzyme-linked immunosorbent assay (ELISA) kit (USA) was used for measuring β-hCG and prolactin.

### Statistical Analysis

T-test and chi-square were used to measure the quantitative and qualitative variables. Receiver operating characteristic curve was used to determine a cut-off value. The cut-off point was set at the highest optimal sensitivity and specificity. The statistical package for social sciences (SPSS) software version 16 (Chicago, IL, USA) was used to analyze the data. A p value less than 0.05 was considered significant.

## RESULTS

There was no significant difference between the two groups regarding demographic characteristics (Table 1). The means of β-hCG, blood urea nitrogen, creatinine and prolactin were 203.1±180.9 mIU/mL, 8.5±6.3 mg/dL, 0.86±0.68 mg/dL, and 69.8±37.9 mIU/mL in the investigation group and 17.4±9.9 mIU/mL, 2.7±1.4 mg/dL, 0.20±0.16 mg/dL, and 10.9±5.6 mIU/mL in the control group. All of the results were significant (p<0.001) (Table 2).

**Figure 1:**
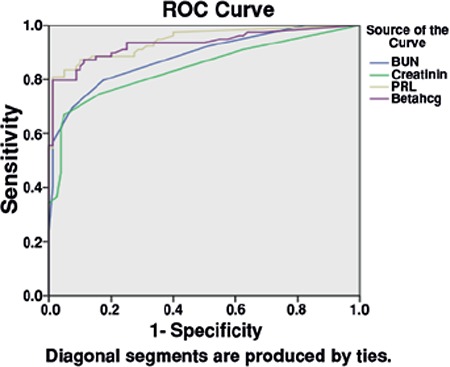
Receiver operating characteristic curve for vaginal beta sub-unit of human chorionic gonadotropin urea, creatinine, prolactin ROC: Receiver operating characteristic, BUN: Blood urea nitrogen, PRL: Prolaktin

Based on the receiver operating characteristic curve, the cut-off point for β-hCG was 20.5 mIU/mL. With that cut-off point, the sensitivity, specificity, positive and negative predictive values for detecting PROM were 87.5%, 86%, 86.4%, and 87.3%, respectively. Also, the cut-off point for blood urea nitrogen was 3.5 mg/dL with 79.7% sensitivity, 82.5% specificity, and 81.8% and 80.4% positive and negative predictive values for detecting PROM. The cut-off point for creatinine was 0.25 mg/dL and it had 74.6% sensitivity, 85% specificity, and 83% and 77.2% positive and negative predictive values for detecting PROM. Finally, the cut-off point for prolactin was 16 ng/mL based with 87.5% sensitivity, 90% specificity, and 90% and 88% positive and negative predictive values (Figure 1). A likelyhood ratio was determined for each diagnostic marker (Table 3).

## DISCUSSION

If PPROM is diagnosed early in pregnancy, many of its adverse effects can be prevented^(1)^. Hence, using biochemical tests and its markers in the vagina has been increased for early diagnosis of ruptured membrane. Various factors such as alpha fetoprotein, insulin-like hormone, prolactin, urea, creatinine and β-hCG,^(5,16,17,18)^ plus alanine transaminase and aspartate transaminase^(19)^. have been suggested and studied. Researchers are still looking for a simple, fast, and easy way to detect membrane rupture that is accessible and non-invasive. Although PAMG-1 is a good choice for detecting PROM, it is not available in most centers and is expensive compared with markers such as prolactin or β-hCG. Thus, some researchers have preferred to find a more convenient diagnostic method.

Bahasadri et al.^(20)^ recorded 93% sensitivity and 84% specificity for β-hCG of vaginal fluid, which is in agreement with our findings. The authors reported that there was more β-hCG in vaginal fluid of women with PPROM than in pregnant women with intact membranes; therefore, it could be a reliable and fast way of detecting membrane rupture. In another study in 2011, Mohamed and Mostafa^(21)^ studied the value of urea, creatinine, and β-hCG of vaginal fluid in detecting rupture of membranes of 298 women. They documented 100% sensitivity and specificity for urea and creatinine and 83% sensitivity and 100% specificity for β-hCG, which are consistent with our findings (Table 4).

In 2009, Taheripanah et al.^(22)^ investigated the diagnostic value of prolactin and β-hCG of vaginal liquid in detecting PPROM. They arrived at 96% sensitivity and 79.4% specificity for prolactin, and 69.3% sensitivity and 69.8% specificity for β-hCG. The authors concluded that although β-hCG could help in detecting membranes rupture, it was not as sensitive and specific as prolactin^(22)^. In our study, when we compared positive likelihood ratios for diagnostic markers, we found prolactin as the marker with the most sensitivity and specificity values. This is in agreement with Taheripanah et al.^(22)^ findings. Kafali and Oksüzler^(23)^ studied urea and creatinine of vaginal liquid with a 12 mg/dL cut-off point for urea and 0.6 mg/dL for creatinine and found that the specificity and sensitivity of both markers was 100%.

In another study, Kariman et al.^(24)^ investigated the diagnostic value of urea and creatinine on vaginal fluid of 179 pregnant women with gestational age of 14 to 42 weeks. For urea, they found 90% sensitivity, 79% specificity, and 83% and 87.5% positive and negative predictive values with a 6.0 mg/dL cut-off point. For creatinine, with a 0.45 mg/dL cut-off point, the authors found 100% sensitivity and specificity. Creatinine had a higher diagnostic value than urea^(24)^. However, creatinine had less diagnostic value in our study, which might have been because of the difference in laboratory analysis methods and cut-off points (Table 4).

In 2004, Buyukbayrak et al.^(2)^ found that prolactin with a 30 µIU/mL cut-off point had 95% sensitivity, 87% specificity, and 87% accuracy, which is consistent with our study. Also, Shahin and Raslan^(5)^ demonstrated lower predictive values for prolactin than in our study. This may be because of the different cut-off points or smaller sample size.

Prolactin and β-hCG have more diagnostic value than urea and creatinine in detecting PPROM, and can be used in suspected cases. These tests are easy and not expensive, and can be used in any medical center. It is suggested that cut-off value for rupture of membranes in pregnancy be determined in different gestational ages in future studies.

## Figures and Tables

**Table 1:**
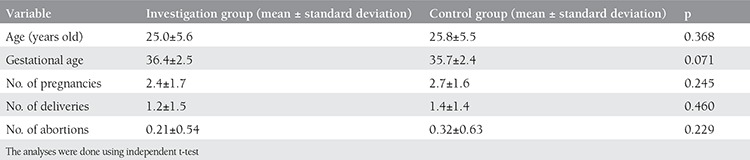
Comparison of the demographic characteristics

**Table 2:**
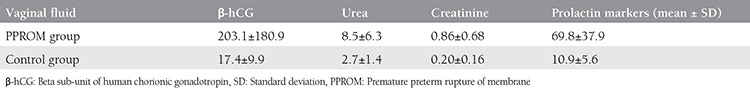
Comparison of means of beta sub-unit of human chorionic gonadotropin urea, creatinine, and prolactin (p<0.001)

**Table 3:**
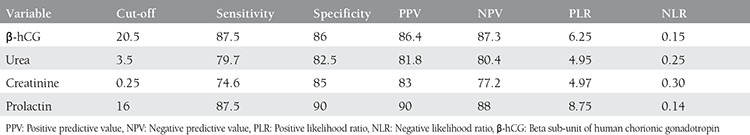
Evaluation of indicators for diagnostic premature preterm rupture of membrane markers

**Table 4:**
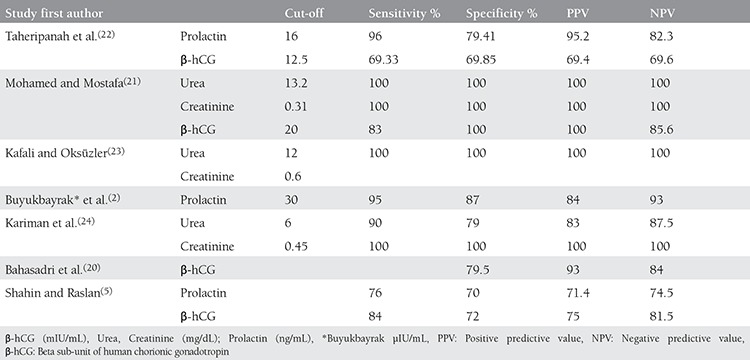
Comparison the values of sensitivity, specificity, positive and negative predictive values for vaginal fluid markers other studies
